# Effect of the Addition of Zeolites on the Resistance to Permanent Deformations of Mastic Asphalt Bridge Pavement

**DOI:** 10.3390/ma18184325

**Published:** 2025-09-16

**Authors:** Lesław Bichajło, Władysław Gardziejczyk, Paweł Gierasimiuk, Krzysztof Kołodziej, Kamil Kowalski, Szymon Malinowski, Tomasz Siwowski, Marta Wasilewska

**Affiliations:** 1 The Faculty of Civil and Environmental Engineering and Architecture, Rzeszow University of Technology, Powstancow Warszawy 12 Street, 35-959 Rzeszow, Poland; krzych@prz.edu.pl (K.K.); k.kowalski@prz.edu.pl (K.K.); siwowski@prz.edu.pl (T.S.); 2 Faculty of Civil Engineering and Environmental Sciences, Bialystok University of Technology, Wiejska 45E Street, 15-351 Bialystok, Poland; w.gardziejczyk@pb.edu.pl (W.G.); p.gierasimiuk@pb.edu.pl (P.G.); marta.wasilewska@pb.edu.pl (M.W.); 3 Faculty of Civil Engineering and Architecture, Lublin University of Technology, Nadbystrzycka 40 Street, 20-618 Lublin, Poland; s.malinowski@pollub.pl

**Keywords:** mastic asphalt, zeolite additives, reduction in the paving temperature, static indentation test, dynamic indentation test, permanent deformation

## Abstract

The mastic asphalt mixture (MA) is one of the first mineral and asphalt mixtures used in history. Its composition and structure allow it (the mixture) to be produced both in industrial conditions (in mineral and asphalt mixing plants) and in field conditions—in mobile boilers (especially when the produced mixture is used to repair damaged surface). The high proportion of the sand fraction makes the mixture highly workable, allowing it to be laid/incorporated without special equipment. MA, however, also has some drawbacks. The asphalt content is higher than in other mixtures, which can make it prone to plastic deformation. Mastic asphalt requires higher processing temperatures than other “hot” mixtures. Mastic asphalt mixtures are installed as road pavement layers and, because of their high density, as the protective layer on roof felt isolation on bridge decks. The high temperature of embedding creates a risk of damaging the roof felt, as its typical temperature resistance is lower than 180 °C, whereas the temperature of the mastic asphalt mixture is higher. The use of zeolites can enable reconciliation of technological requirements of mastic asphalt and asphalt roof isolation. The mixes MA 8 and MA 11 containing 0 and 5% of two types of zeolites and asphalt binders 35/50 or elastomer-SBS-modified asphalt binder PMB 25/55-60 were used in the research. Laboratory tests revealed that the addition of a 5% amount of zeolite by asphalt mass makes it possible to reduce the mastic asphalt laying temperature by up to 30 °C, which seems to be very important from ecological, economical, and pavement durability points of view.

## 1. Introduction

Mastic asphalt (MA) has become a popular choice for bridge pavements because of its many advantages, including easy application, excellent waterproofing, and high durability [1,2]. MA typically consists of a blend of paving-grade bitumen and often a modifier (forming a modified binder), which is then mixed with fine aggregate (filler) to produce a “mastic”, also known as composite bitumen or composite asphalt. Coarse aggregate is then added to complete the mixture. Thanks to its high fluidity and self-leveling properties, mastic asphalt does not require compaction during paving [3]. Applying one or two layers of MA provides exceptional protection for the bridge deck against water and de-icing agents. This is due to the mixture’s low permeability, with an air void content of less than 1% [4]. When compared to standard asphalt concrete (AC) or stone mastic asphalt (SMA) pavements, MA pavements have a longer service life and greater fatigue resistance. This is because the high bitumen content makes the mixture highly viscoelastic and less prone to cracking [3,5,6,7].

Mastic asphalt is often characterized by the symbol “3H”: high binder content (6.5–10%), high filler content (20–40%), and high laying temperature (220–240 °C) [1]. These properties can significantly affect the resistance to permanent deformation (high binder content) as well as the environmental harmfulness (high laying temperature). Therefore, in practice, various binder additives are used to reduce the negative effects of mastic asphalt properties. 

To make mastic asphalt resistant to permanent deformation, products that “harden” the bitumen are used, such as waxes, polymers, and natural asphalts [5,8,9]. In the latter case, Trinidad Lake Asphalt (TLA) is applied [10,11]. The most significant advantages of using these bitumen modifiers are optimized structural properties of the binder (leading to lower brittleness and greater resistance to aging), improved adhesion of the binder to mineral aggregates, enhanced workability and compaction of the mixture, and, ultimately, greater resistance to permanent deformation and fatigue. High-quality MA pavements containing modified binders have been successfully used for many years on hundreds of bridges worldwide [12,13,14].

However, the high mixing and working temperatures of mastic asphalt can pose a significant problem. Polymer-modified MA products, in particular, may require working temperatures up to 230 °C, depending on the laying conditions [15]. While the working temperature (including production, laying, and paving) must be high enough to ensure good workability (Lueer fluidity should not be larger than 20 s [16]), excessively high temperatures can negatively affect the binder’s properties or cause the polymer to degrade. Working at these high temperatures is also energy-intensive and releases more bituminous fumes and carbon dioxide compared to conventional hot-mix asphalt operations [17]. This has become a growing concern as stricter regulations on working temperatures and emissions have been implemented [18]. The high temperature of MA laying also releases a significant amount of heat into the atmosphere, which contributes to the urban heat island effect and poses health risks to workers and residents [19]. Since MA construction sites are often limited in space, manual paving is common, making the emissions a direct health risk to workers. Reducing the temperature of MA mixtures would decrease costs, energy consumption, and emissions of harmful particulate matter throughout the product’s life cycle.

In recent years in Poland, the high working temperature of MA pavement has caused major durability issues for bridge waterproofing [6]. Bridge pavements in Poland typically consist of a wearing course and a protective layer placed over the waterproofing. MA mixes are commonly used for the protective layer to ensure its tightness. The high level of waterproofing required for bridge pavements—achieved through a low air void content and high binder content in the protective layer—further increases the susceptibility of these layers to strain, especially at high working temperatures. Research by authors in [20,21] investigated the thermal effects on bridge deck materials and bituminous pavements during construction. The research was prompted by a need to understand why blistering of bituminous waterproofing membranes and asphalt protective layers is frequently observed on bridge decks. The studies confirmed that technological temperatures significantly impact these materials and that, under these conditions, it is possible for reactions between concrete slab components to produce gaseous products.

While there is extensive knowledge on various techniques for producing lower-temperature MA mixtures, there is insufficient data on how these techniques affect the final performance of the mastic asphalt. Recently, many researchers have focused on the most promising methods to reduce the laying temperature of MA mixtures, both in the lab and in the field. The most promising techniques involve using foamed bitumen, adding waxes to the bitumen, using bio-oil (e.g., castor oil combination with montmorillonite nanoparticles), and adding zeolites to the mixture [22,23,24,25,26]. This paper focuses on the last technique. Zeolites are crystalline, hydrated aluminum silicates that can store a large amount of water within their crystal structure. When added to hot bitumen just before mixing with aggregate, the zeolites release water vapor, creating a continuous foaming effect. This allows for a reduction in production and compaction temperatures while maintaining sufficient workability. Zeolites, including natural and synthetic types, are currently used in warm mix asphalt (WMA) technologies. The review [27] presents the results of studies on WMA technology, including the effects of zeolite addition on asphalt properties, as well as related environmental, economic, and technological benefits. The authors state that zeolites, depending on their origin, can be used in amounts ranging from 0.25% to 1.0% by weight of the asphalt mixture. Their use reduces binder viscosity by releasing water contained in the pores, which simultaneously improves the workability of the mixture. This allows the production temperature of the asphalt mixture to be lowered by approximately 30 °C. Such a temperature reduction leads to decreased greenhouse gas emissions and reduced production costs. No adverse effects of zeolite addition on the mechanical properties of the mixtures were observed. Only moisture resistance may deteriorate, which can be prevented by adding hydrated lime. In the study [28], the possibility of decreasing the compaction temperature of AC was analyzed. The additives applied included natural zeolite—clinoptilolite—and synthetic NaP1 zeolite obtained from fly-ashes. In compatibility tests in a gyratory compactor, the optimal additive of zeolite was established, which included 1% clinoptilolite, 0.5% NaP1, and 0.4% of both zeolites soaked with water w/m. In turn, in the paper [29] the authors present the properties of an SMA LA (stone matrix asphalt Lärmarmer) mixture based on the polymer-modified binder PMB 45/80-55, formed by the addition of the same two zeolites. Adding zeolites reduced the production temperature by as much as 15–20 °C. The addition of zeolites did not significantly affect the resistance to permanent deformation, the water permeability, or the mass loss. The mechanism of the technological temperature reduction is characterized in Section 2.2.

The paper presents the results of the authors’ research on the use of the same zeolites as above to reduce the laying temperature of two MA mixtures produced with two different types of bitumen. The performance of the MA in terms of permanent deformation is compared with that of a reference MA without additives. Several aspects have been studied: type of bitumen, type of zeolite, grading curve of aggregate, and their effect on the permanent deformation using static and dynamic indentation methods. This study includes the investigation of two zeolite additives to the non-modified and modified asphalt binder and associated foaming phenomena. Two zeolites differ in their crystalline structures, i.e., one was Na-P1 synthetic zeolite and the second was clinoptilolite natural zeolite. The amount of zeolite added to the asphalt binder was a constant 5% by asphalt mass. This is because tests conducted by other authors showed that using such an amount of additives can reduce the technological temperature of the other types of asphalt mixes by up to 30 °C [20,21,22]. As Sengoz et al. have revealed in [30], the addition of zeolites has changed the viscosity of asphalt binder with natural and synthetic zeolite, decreasing and then increasing with zeolite content. The minimum viscosity they noticed was at a 5% zeolite content. More significant changes of complex stiffness modulus were observed when the natural zeolite content exceeded 5%, when no significant variation was achieved with synthetic zeolite additives. The literature provides numerous studies on the use of zeolites in rolled asphalt mixtures, e.g., in AC or SMA, but does not address the addition of zeolites to mastic asphalt mixture, which is a relatively rarely used mixture [27,28,29,30]. Taking the above into account, we used the amount of additive suggested in the literature for rolled mixtures. Determining its permissible content will be the subject of other studies by the authors.

## 2. Materials and Methods

### 2.1. Mastic Asphalt Mixtures

**Figure 1:**
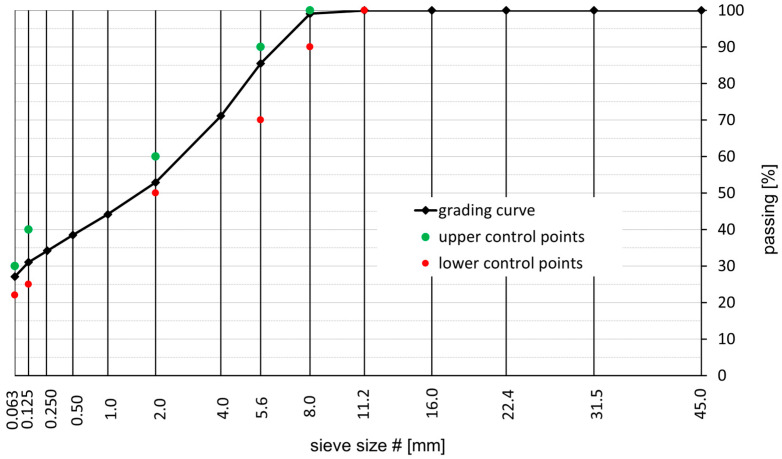
Grading curve of the tested MA 8 mixture.

**Figure 2:**
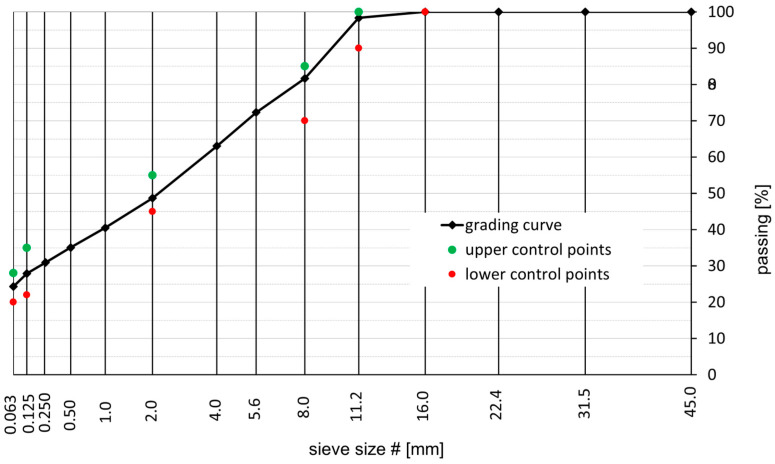
Grading curve of the tested MA 11 mixture.

Two MA mixtures were chosen for the study: the MA 8 mixture and the MA 11 mixture. These two mixtures are used most commonly as constituents of bridge pavement in Poland and thus were designed according to the Polish specification in this regard [18]. The Trzuskawica limestone powder (Trzuskawica S.A., Nowiny, Poland) was used as a filler, 0/2 fine aggregate and 2/5, 4/8, and 8/11 gabbro aggregate (KSS Bartnica, Braszowice, Poland) constituted coarse aggregate in both mixtures. The grain size curves of the MA mixtures tested are shown in Figure 1 and Figure 2, for MA 8 and MA 11, respectively.

Road bitumen 35/50 (PKN Orlen, Plock, Poland) and SBS modified bitumen PMB 25/55-60 (PKN Orlen, Plock, Poland) were used as binders. Table 1 shows the properties of both the bitumen used in the tests. The amount of asphalt was assumed to be higher than the amount of B_min_, according to [18], i.e., 6.5% in relation to the mass of the MA mixture.

**Figure 3:**
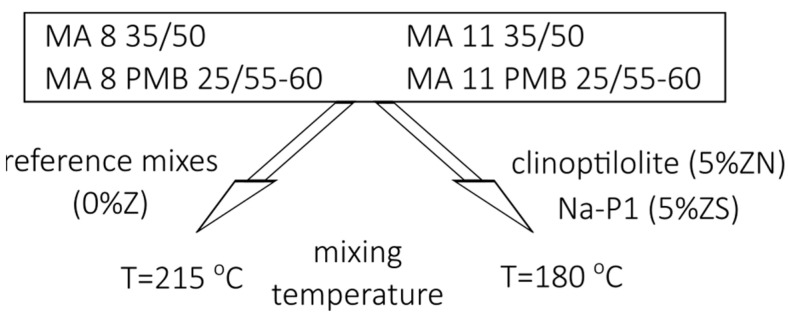
The MA mixtures tested.

Using the materials presented above (two aggregate sizes and two bitumen types), four different MA mixtures were prepared for testing, with the addition of natural and synthetic zeolites. To check the effect of zeolite additives, all four MA mixtures were supplemented with 5% of the natural zeolite clinoptilolite (Sokyrnytsya, Transcarpathian region, Ukraine) (designated as 5%ZN) and 5% of the synthetic zeolite Na-P1 (Lublin University of Technology, Lublin, Poland) (designated as 5%ZS). The four reference MA mixtures without zeolites were designated as 0%Z. Samples of the mixture without zeolite were molded at 215 °C, while those with zeolite were molded at 180 °C. The mixtures tested in this study are shown schematically in Figure 3.

### 2.2. Zeolites

Clinoptilolite is a natural zeolite that is easily available in some regions of Poland, and its proven properties allow it to be used in mastic asphalt. The chemical composition of the clinoptilolite used in the study was determined by the X-ray Fluorescence (XRF) method using a Philips PW 1404 spectrometer equipped with a Cr-Au double anode with a maximum power of 3 kW as excitation source (Table 2) 2θ (Panalytical, Almelo, The Netherlands). The main components of the clinoptilolite are SiO_2_ and Al_2_O_3_, found in amounts of ≈77% *w*/*w* and ≈11% *w*/*w*, respectively, so that the SiO_2_/Al_2_O_3_ ratio in the material was ≈6.8. In addition, K_2_O, CaO, and Fe_2_O_3_ are present in the clinoptilolite in amounts ranging from ≈2.5% *w*/*w* to ≈4% *w*/*w*, and in trace amounts of <1% *w*/*w* MgO, TiO_2_, MnO, Rb_2_O, SrO, ZrO_2_, Ag_2_O, and BaO.

**Figure 4:**
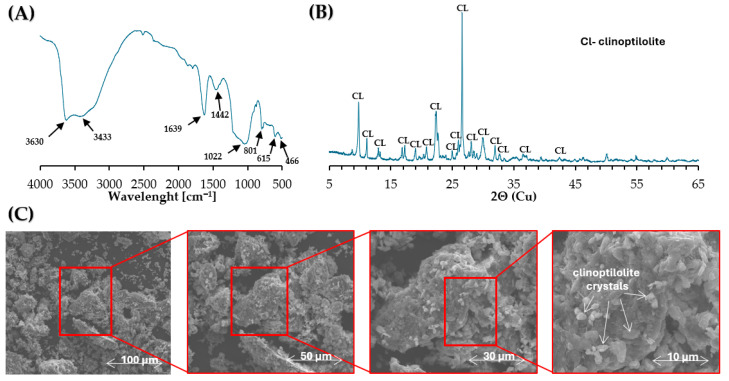
FTIR spectra (**A**), XRD pattern (**B**), and SEM images of clinoptilolite (**C**).

Fourier transform infrared (FTIR) spectroscopy analysis was used to perform the chemical analysis of the clinoptilolite used in this study. Chemical bonds were investigated using Diffuse Reflectance Spectra (FTIR/DRS) recorded on a Nicolet 380 spectrophotometer from Thermo Scientific (Thermo Scientific, Waltham, MA, USA). The spectrum of clinoptilolite was recorded in the range of 4000–500 cm^−1^ at room temperature, with a resolution of 4 cm^−1^ and a mirror speed of 2.5 kHz without smoothing function. During the FTIR spectrum recording of clinoptilolite, 1024 scans were collected to ensure an appropriate signal-to-noise ratio. The obtained clinoptilolite spectrum was then normalized by comparing the obtained spectrum with the background spectrum (spectrum of dried and smeared KBr). The FTIR spectrum of clinoptilolite, shown in Figure 4A, shows bands at 3630 cm^−1^ and 3433 cm^−1^ wave numbers from the stretching vibrations of the -OH groups connecting silicate and/or aluminum tetrahedra. The first of the above may indicate the presence of adsorbed H_2_O molecules in the structure of the clinoptilolite. It is also indicated by the presence of a band located at 1639 cm^−1^ corresponding to H-O-H scissor vibrations [35].

The structure of the clinoptilolite in the FTIR spectrum (Figure 4A) can be seen from the bands located at wavelengths of 1022 cm^−1^ and 615 cm^−1^, corresponding to the antisymmetric and symmetric vibrations of the tetrahedra (T-O-T, where T = Si or Al) building it. The intensities of the bands of the key structures that build the clinoptilolite, observed on the FTIR spectrum, also find reference in the chemical composition determined by the XRF method (Table 2).

The mineral composition of the clinoptilolite was determined by XRD. The analysis was carried out by the powder method using a Panalytical X’pert PROMPD X-ray diffractometer with a PW 3050/60 goniometer in the angular range of 5–65 2θ (Panalytical, Almelo, The Netherlands). A Cu copper lamp (CuKα = 0.154178 nm) was used as the X-ray source. The identification of mineral phases was based on the 2010 edition PDF-2 database, formalized by JCPDS/ICDD. The obtained XRD pattern is shown in Figure 4B and shows peaks originating from the crystalline phases that build up clinoptilolite.

The morphology of the clinoptilolite was examined using a Quanta 250 FEG scanning electron microscope (SEM) (FEI Co., Hillsboro, OR, USA). The microphotographs shown in Figure 4C confirm the characteristic reticular flake-like structure that forms numerous macro-pores and well-defined clinoptilolite crystals.

Na-P1 zeolite is a synthetic material created as an equivalent to natural zeolite. Zeolite Na-P1 represents a framework similar to GISmondine (GIS), where eight-membered channels of diameter 3.1 × 4.5 Å and 2.8 × 4.8 Å are made up of two four-membered aluminosilicate rings. The detailed characteristics of the synthetic zeolite used in the research are provided in the article [36].

In the conducted study, both zeolites were saturated with water at 75% *w*/*w* prior to use. After heating to the mixing temperature, the water accumulated in the pores of the molecules gradually releases. As a result, the viscosity of the asphalt is reduced, which allows for the production and incorporation of MA at a lower temperature. During the cooling of the binder, the micro-bubbles of steam condense, which causes the viscosity of the binder to increase again to the original value, and the binder and the produced MA regain their initial (original) properties [27].

### 2.3. Testing Methods

In order to assess the resistance of the MA to permanent deformation, static and dynamic indentation tests were used. The static indentation was conducted according to EN 12697-20 [37] using standardized 70.7 mm cubic specimens. The load was transferred to the samples using an indentor pin with a circular base (a cylindrical piston with a contact surface area of 500 mm^2^) and the penetration of the indentor into the sample (indentation) was measured after 30 and 60 min. The load was applied for some time, starting with the initial load of 25 N and then, after 10 min of preloading, increasing the load by 500 N, thus giving the total test load of 525 N. The constant temperature of 40 °C was maintained during the test, when the sample remained submerged in water. The values registered as the results of the test included indentation of the piston after 30 min and the increase in the indentation after an additional 30 min of constant loading. For each series, six test samples were taken.

The dynamic indentation test was carried out in accordance with the EN 12697-25 [38] and EN 13108-20 [39] standards, at a temperature of 50 °C, on cylindrical samples with a diameter of 150 mm and a depth of approximately 60 mm. Before the dynamic indentation test, the samples were seasoned for 4 h at the test temperature and then subjected to a static preload of 10 kPa. The load was transferred using a steel cylinder with a surface area of 2500 mm^2^ (punch diameter 56.4 mm), in a cyclical manner, with the following parameters:

- Maximum load value: 0.875 kN (corresponds to a sample load of 0.35 N/mm^2^);
- Minimum load value: 0.20 kN (corresponds to a sample load of 0.08 N/mm^2^);
- Load duration: 0.2 s;
- Rest time: 1.5 s;
- Cycle duration: 1.7 s;
- Load curve shape: half-sine.

**Figure 5:**
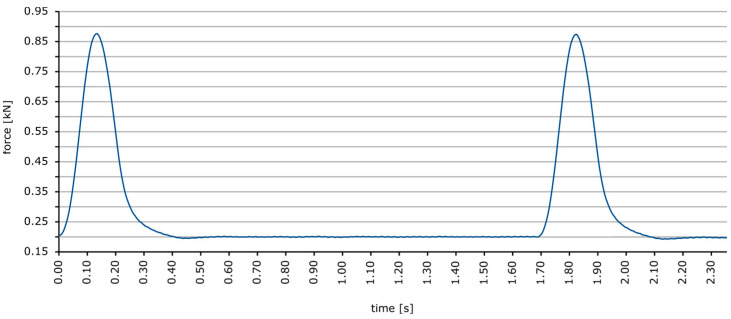
Load pattern used in dynamic indentation test.

**Figure 6:**
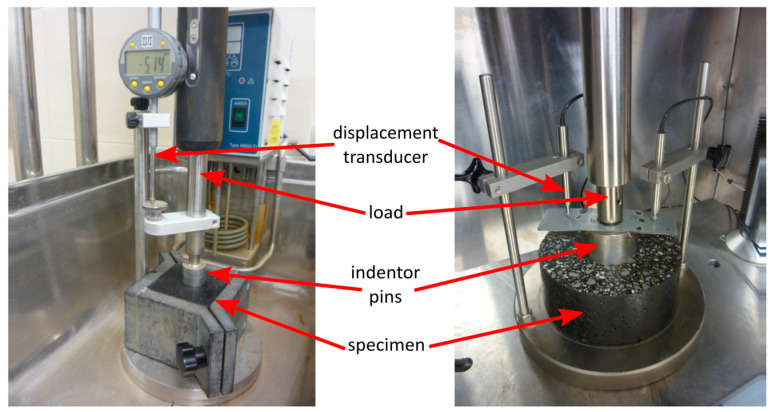
Equipment used in static and dynamic indentation test.

The load pattern is shown in Figure 5. The results were read after 2500 and 5000 dynamic load cycles. The plunger in the sample was assumed to be the average of three displacement values: two from Linear Variable Differential Transformer (LVDT) sensors based on a plate attached to the pin and one from the actuator displacement sensor. Four test samples were made for each series. Figure 6 shows the equipment used in the study.

The obtained test results were subjected to statistical analysis consisting of checking the normality of the distribution of results, rejecting gross errors, and calculating the mean values and measurement uncertainties (type A method, by statistical analysis of the series of observations). Outliers were rejected using the Grubbs method. Comparisons of the obtained results depending on the factor were performed using Student’s *t*-test. In the calculations, the significance level of α = 0.05 was assumed, which means that only 5% of the results may fall outside the designated measurement uncertainty range.

## 3. Results

### 3.1. Static Indentation Test

**Figure 7:**
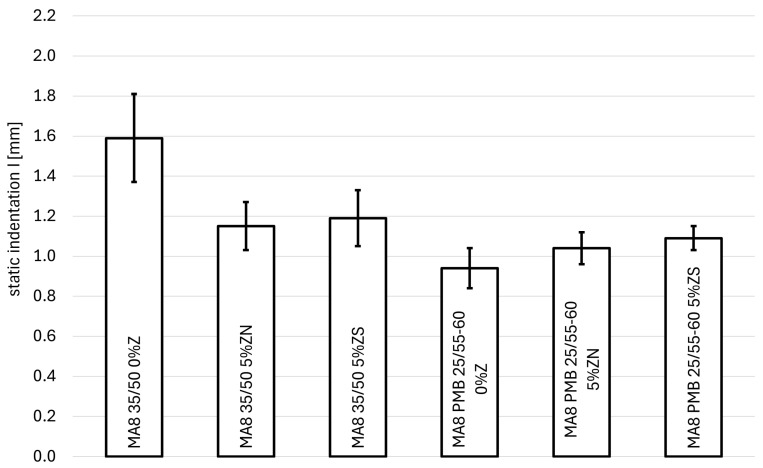
Dependence of static indentation on the type of binder and the amount of zeolite addition for the MA8 mixtures.

**Figure 8:**
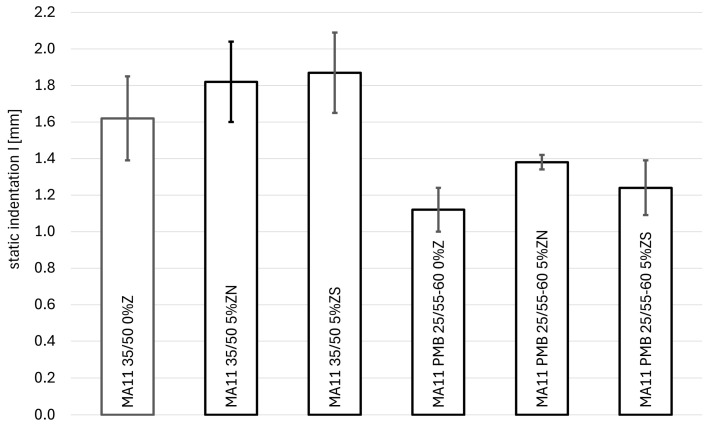
Dependence of static penetration on the type of binder and the amount of zeolite addition for the MA11 mixtures.

The results of the static indentation test of resistance to permanent deformation are presented in Table 3, Figure 7 (for MA 8) and Figure 8 (for MA 11). Table 4 presents the increase in static indentation between 30 and 60 min loading.

### 3.2. Dynamic Indentation Test

**Figure 9:**
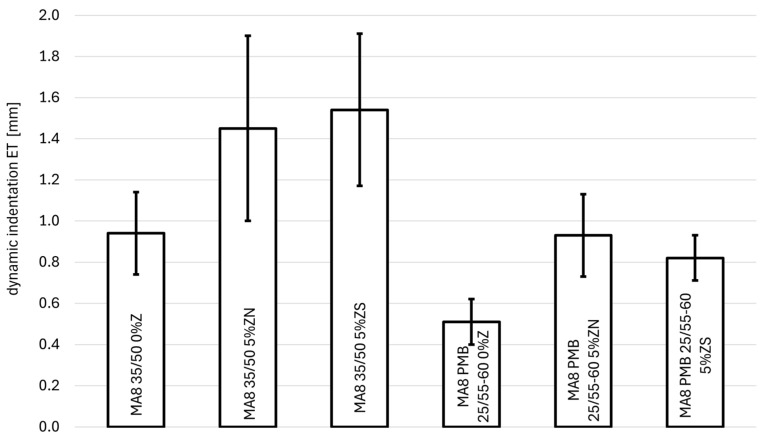
Dependence of dynamic penetration on the type of binder and the amount of zeolite addition for the MA8 mixtures.

**Figure 10:**
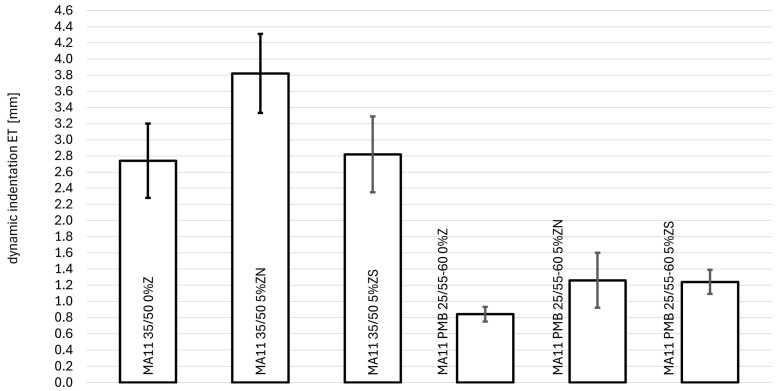
Dependence of dynamic penetration on the type of binder and the amount of zeolite addition for the MA11 mixtures.

The results of the dynamic indentation test of resistance to permanent deformations are presented in Table 5, Figure 9 (for MA 8) and Figure 10 (for MA 11). Table 6 presents the increase in dynamic indentation between 2500 and 5000 cycles.

## 4. Discussion

### 4.1. Resistance to Permanent Deformation in Static Indentation Test

For the MA 8 mixture with 35/50 bitumen, a decrease in the static indentation value of 0.44 mm and 0.40 mm can be observed in the MA with clinoptilolite and synthetic zeolite Na-P1, respectively. In both cases, the zeolites significantly influenced the resistance to permanent deformation of the MA 8 mixture, i.e., the statistical significance in the case of natural zeolite is *t*(7.76) = 4.53, *p* < 0.05 and for synthetic zeolite is *t*(8.61) = 3.92, *p* < 0.05. However, the difference between the penetration values obtained for both zeolites is very small (0.04 mm) and is not statistically significant (*t*(9.72) = 0.583, *p* = 0.573). For both zeolites, the increase in deformation between the 30th and 60th minute of the testing is 0.12 mm and represents a decrease of 0.04 mm compared to the mixture without these additives. It is not possible to clearly indicate which of the used zeolites has a better effect on the tested mixture. In both cases, this effect is at a similar level.

For the MA 8 mixture with PMB 25/55-60 bitumen, an increase in the static indentation value can be observed; thus, the mixture shows slightly weaker resistance to deformation. However, the increase in static indentation value is statistically insignificant in the case of the addition of natural zeolite (*t*(9.69) = 2.07, *p* = 0.07); thus, this additive does not negatively affect the permanent deformation of the MA 8 mixture. In the case of synthetic zeolite, this result is statistically significant (*t*(7.38) = 3.38, *p* = 0.01). However, when comparing the static indentation values, it can be observed that the increase in penetration is only 0.15 mm for PMB 25/55-60 bitumen. In comparison to the increase in deformation, in the case of using natural zeolite, a small decrease in the increase in deformation was observed compared to the reference mixture, and in the case of synthetic zeolite—a small increase. The differences are on the order of 0.01 mm. It can be concluded that natural zeolite is a slightly better additive in terms of resistance to permanent deformations of the MA 8 mixture with PMB 25/55-60 bitumen.

For the MA8 reference mixture, changing the type of bitumen from 35/50 to the modified PMB 25/55-60 significantly affected the static indentation value obtained; the difference is 0.65 mm (*t*(7.01) = 7.00, *p* < 0.05). As expected, the mixture with modified PMB 25/55-60 bitumen shows greater resistance to permanent deformation. A similar situation occurs in the case of the use of a zeolite additive. Both tested zeolites affected the deformation properties of the MA8 mixture in a similar way, and there are no significant differences between them. In the case of 35/50 road bitumen and PMB 25/55-60, the differences between them are statistically insignificant (*t*(9.71) = 0.58, *p* = 0.57 for 35/50 bitumen and *t*(8.10) = 1.13, *p* = 0.29 for PMB 25/55-60 bitumen). However, the mixtures with the addition of synthetic zeolite show a slight deterioration in resistance to permanent deformations. On this basis, it can be concluded that the best results in terms of resistance to permanent deformations are shown by the MA8 PMB 25/55-60 mixture with natural zeolite.

For the MA11 mixture with 35/50 bitumen, the test shows an increase in the penetration value of 0.20 mm for natural zeolite and 0.25 mm for synthetic zeolite. However, the results obtained from the *t*-Student test (*t*(9.97) = 1.61, *p* = 0.139 for natural zeolite and *t*(9.97) = 2.00, *p* = 0.07 for synthetic zeolite) suggest that this change does not significantly affect the resistance to permanent deformation of this mixture. Thus, it is not possible to indicate which of the zeolites is better in these terms. The difference in the penetration value between them, equal to 0.05 mm, is statistically insignificant (*t*(9.99) = 0.409, *p* = 0.691) and is the result of the natural variability of the samples and not the type of additive used. In the case of increased penetration, a slight deterioration in resistance to deformation can be observed, with the synthetic zeolite Na-P1 showing to be better. Here, the increase in the deformation increase value is smaller than in the case of natural zeolite.

For the MA11 mixture with PMB 25/55-60 bitumen, we also observed an increase in the static indentation value. In the case of natural zeolite, this is an increase of 0.26 mm (23%), and for synthetic zeolite, 0.12 mm (11%). In the case of natural zeolite, this difference is statistically significant compared to the reference mixture, as evidenced by the results of the *t*-Student test (*t*(5.47) = 3.93, *p* < 0.05). However, in the case of the synthetic zeolite, this difference is not statistically significant (*t*(9.87) = 1.40, *p* = 0.19). Comparing both zeolite additives with each other, it can be stated that the difference between them is statistically insignificant (*t*(5.60) = 2.32, *p* = 0.06). It is worth noting that the obtained result is on the borderline of being considered statistically significant, so its interpretation should be approached with caution. Analyzing the value of the static indentation increase, it can be stated that the addition of zeolite improves this feature in both cases, because the deformation increase decreased: for natural zeolite by 0.01 mm, and for synthetic zeolite by 0.03 mm. On this basis, it can be stated that the use of synthetic zeolite Na-P1 would be a better additive.

For the MA11 reference mixture (without zeolites), the change in the type of bitumen significantly affected the obtained penetration value (*t*(8.94) = 4.51, *p* = 0.001). The use of modified bitumen caused a decrease in the static indentation value by 0.5 mm, which is 31% of the penetration value for the mixture with the 35/50 road bitumen. A similar situation occurs in the case of the use of zeolite additive. For a given type of zeolite, changing the type of bitumen from road bitumen to a modified one results in improved resistance to permanent deformations. For clinoptilolite, a decrease in the static indentation value of 0.44 mm (i.e., 24%) can be observed, and for the synthetic zeolite Na-P1 the reduction in the static indentation value was 0.63 mm, which is a decrease of 34% compared to the mixture with road asphalt. For the tested MA 11 mixtures, the best is the MA 11 PMB 25/55-60 mixture with the addition of synthetic zeolite Na-P1.

### 4.2. Resistance to Permanent Deformation in Dynamic Indentation Test

For the MA8 mixture with 35/50 bitumen, a deterioration in the resistance to permanent deformation assessed by the dynamic indentation test can be observed with an addition of 5% zeolite. For clinoptilolite, the dynamic indentation value after 2500 cycles increased by 0.51 mm (54%), and for synthetic zeolite Na-P1 the deformation increased by 0.60 mm (64%). In both cases, the addition of zeolites to the MA 8 mixture significantly affected the resistance to permanent deformation (the results of the *t*-Student test are *t*(4.80) = 3.45, *p* = 0.02 for natural zeolite and *t*(4.58) = 5.36, *p* = 0.004 for synthetic zeolite, respectively). In this case, natural zeolite–clinoptilolite had a lesser effect on the deterioration of the properties of the tested mixture. This is also confirmed by the results of the deformation increase between the 2500/5000 test cycles. For both analyzed zeolites, the deformation increase increased by 0.13 mm for natural zeolite and by 0.20 mm for synthetic zeolite. It can also be observed that natural zeolite–clinoptilolite had a smaller effect on the deterioration of the properties of the MA8 35/50 mixture.

Analogous results were obtained using modified bitumen PMB 25/55-60. Here, too, reducing the addition of zeolites worsened its resistance to permanent deformations. For natural zeolite, dynamic penetration increased by 0.42 mm (82%), and for synthetic zeolite it increased by 0.31 mm (61%), compared to the reference mixture without zeolites. Both obtained results are statistically significant (the results of the *t*-Student test are *t*(4.67) = 5.80, *p* = 0.002 for natural zeolite and *t*(4.93) = 7.25, *p* < 0.05 for synthetic zeolite, respectively). In this case, the synthetic zeolite Na-P1 proved to be slightly better, causing a smaller increase in permanent deformation. This is also confirmed by the results of the deformation increase between the 2500/5000 test cycles. For natural zeolite, the deformation increase increased by 115% (0.08 mm), and for synthetic zeolite, the penetration increase increased by 86% (0.06 mm).

Changing the type of bitumen from road bitumen to modified bitumen affected the resistance of the mixture to permanent deformation. For the reference mixture, the dynamic indentation value decreased by more than half. A similar decrease in the dynamic indentation value occurred in the case of synthetic zeolite. However, in the case of natural zeolite, a reduction in penetration values by more than 65% can be observed. Comparing both zeolites with each other, depending on the type of binder, it cannot be clearly stated that there is a difference between them in terms of the effect on the deterioration of resistance to permanent deformations. Comparison of this effect using Student’s *t*-test does not show any significant differences between the zeolite additives tested: *t*(4.85) = 0.61, *p* = 0.567 for 35/50 bitumen and *t*(3.87) = 1.57, *p* = 0.193 for PMB 25/55-60 bitumen. Based on the above analyses for the MA 8 mixture, it cannot be clearly stated which of the tested zeolites has a more beneficial effect on the obtained values of resistance to permanent deformations. Both zeolites affect the properties of the tested MA 8 mixtures in a similar way.

For the MA 11 mixture with 35/50 bitumen, the use of zeolites had a different effect on the dynamic indentation values obtained and thus on the resistance to permanent deformation. For natural zeolite, dynamic penetration increased by 1.08 mm (an increase of 39% compared to the reference mixture), and for synthetic zeolite, the increase was only 0.08 mm (an increase of less than 3%). In the case of clinoptilolite, the obtained results suggest a significant effect of the additive on the test results (*t*(4.90) = 5.80, *p* = 0.002), while in the case of the use of synthetic zeolite Na-P1, this effect is statistically insignificant (*t*(4.95) = 0.44, *p* = 0.676). The increase in deformation looks similar. In the case of natural zeolite, an increase in the value of the deformation increase by 0.25 mm was observed, while for synthetic zeolite, this increase was only 0.02 mm. On this basis, it can be stated that the best effects are achieved with the use of synthetic zeolite Na-P1.

In the case of the MA 11 mixture with the modified bitumen PMB 25/55-60, a deterioration in resistance to permanent deformations was observed with the addition of zeolite. However, in this case, the effect of both zeolites was similar. The addition of natural zeolite increased the penetration value by 0.42 mm and for synthetic zeolite by 0.40 mm. This is also confirmed by the results of the penetration increase between the 2500/5000 test cycles. For both zeolites, the increase in the penetration increase value is almost identical and amounts to approx. 0.08 mm. In both cases, the results obtained from the increase in the penetration value are statistically significant (*t*(3.41) = 3.78, *p* = 0.03 for natural zeolite and *t*(4.99) = 7.39, *p* < 0.05 for synthetic zeolite).

Changing the type of bitumen also improves resistance to permanent deformations assessed by the dynamic indentation test. As expected, the use of modified bitumen improved the resistance to permanent deformations of the MA11 mixture. In this case, the penetration reduction was 1.90 mm, i.e., a reduction of almost 70%. A similar reduction occurred in the case of natural zeolite; a reduction in the penetration value of 2.56 mm, i.e., 67%, was observed, while in the case of synthetic zeolite, the reduction in penetration was 1.58 mm, i.e., 56%. Comparing both zeolites with each other, it can be stated that depending on the type of asphalt, they have a different effect on the behavior of the mixture. In the case of road bitumen 35/50, the effect of the type of zeolite is statistically significant (*t*(5.99) = 4.75, *p* = 0.003) and here it can be clearly indicated that synthetic zeolite Na-P1 has a lesser effect on the deterioration of resistance to permanent deformations of the mixture. In the case of polymer-modified bitumen, it is not possible to clearly determine which of them has a smaller effect on reducing resistance to permanent deformations. Analysis of the results using the *t*-Student test does not show any significant differences between the zeolites tested (*t*(4.06) = 0.19 *p* = 0.857). The above analyses show that in the study of the MA11 mixture with 35/50 bitumen, it is more beneficial to reduce the temperature using the synthetic zeolite Na-P1. It has the smallest effect on reducing resistance to permanent deformations. In the case of PMB 25/55-60 bitumen, both zeolites affect the behavior of the tested MA 11 mixture in a similar way, and it is impossible to indicate which of them has a more beneficial effect on the obtained results.

### 4.3. Effect of the Test Method

Both test methods similarly classify the mixtures of mastic asphalt in terms of resistance to permanent deformations. Only in the case of the MA 8 mixture with 35/50 bitumen was a decrease in the penetration value observed after the use of zeolite additive in the static indentation test, while for the dynamic indentation test, in all cases the penetration value increased, resulting in a reduction in resistance to permanent deformations. The dynamic indentation test is recommended for hard mastic asphalt mixtures with static indentation values below 2.5 mm. In the case of penetration testing using the static indentation method, although the guidelines are met, some mixtures are excessively rutted. The dynamic method reflects the actual rutting conditions of the tested mixtures to a greater extent; therefore, its use allows for a better assessment of the tested mixtures. For the MA 8 mixture with road bitumen, it can be stated that both methods classify the tested mixture differently. In the case of static penetration, an improvement in resistance to permanent deformations was observed, while in the dynamic indentation test, it deteriorated. For the MA 8 mixture with modified bitumen, both static and dynamic penetration shows a deterioration in resistance to permanent deformations. Differences occur in the case of the zeolite used. For static penetration, natural zeolite—clinoptilolite—is a better additive, while for dynamic penetration—synthetic zeolite Na-P1.

In the case of the MA11 mixture with 35/50 bitumen and PMB25/55-60-modified bitumen, both test methods indicate a deterioration in resistance to permanent deformations. However, differences can be observed depending on the type of zeolite used. For 35/50 bitumen, natural zeolite is better in the static test, while synthetic zeolite is better in the dynamic test. In the case of PMB 25/55-60-modified bitumen, both test methods indicate that synthetic zeolite Na-P1 is better. As mentioned earlier, the differences between the analyzed zeolites are statistically insignificant, so it is possible to generalize the conclusions that both test methods classify the zeolites in a similar way.

According to Polish requirements WT-2 2014 [18], the penetration of a static indentation of a mastic asphalt mixture should be within the range of 1–3 mm, while the increase in penetration should be less than 0.6 mm. Almost all tested mixtures meet this requirement; only the reference mixture MA 8 with PMB 25/55-60 bitumen shows penetration slightly below the lower limit. The analyzed mastic asphalt mixtures with the addition of zeolite, despite the deterioration of resistance to permanent deformations, meet these requirements. In the case of dynamic indentation, there are no Polish requirements in this area. However, one can refer to the Swiss requirements [40], for which the required dynamic penetration value after 2500 cycles for a mastic asphalt mixture for a surface loaded with light traffic should not exceed 5.0 mm, and the increase should not exceed 2.2 mm. In the case of heavy traffic, the dynamic penetration should not exceed 2.5 mm and the increase in this penetration should not be greater than 0.8 mm. The analyzed mixtures mostly meet the requirements for heavy traffic; only the MA11 mixture with 35/50 bitumen does not meet this requirement, but it is suitable for light traffic. The addition of zeolite did not deteriorate the properties of the mixture to such an extent that it did not meet the requirements for the reference mixture. On this basis, it can be concluded that the use of zeolites to lower the laying temperature of the MA mixture does not significantly worsen its resistance to plastic deformation (however, in most cases the effect is not statistically significant) and does not pass the limits included in technical specifications [18].

## 5. Conclusions

The paper presents the results of the authors’ research on the effect of the addition of two zeolites on the resistance to permanent deformations of MA bridge pavement. Two MA mixtures (MA 8 and MA 11) with two different binders (road and modified) supplemented with two types of zeolite (natural and synthetic) were tested in static and dynamic penetration tests to assess the resistance of MA mixtures to permanent deformation. The authors state that zeolites, depending on their origin, can be used in amounts ranging from 0.25% to 1.0% by weight of the asphalt mixture. Their use reduces binder viscosity by releasing water contained in the pores, which simultaneously improves the workability of the mixture. This allows the production temperature of the asphalt mixture to be lowered by approximately 30 °C. Such a temperature reduction leads to decreased greenhouse gas emissions and reduced production costs. No adverse effects of zeolite addition on the mechanical properties of the mixtures were observed. Only moisture resistance may deteriorate, which can be prevented by adding hydrated lime.

The following detailed conclusions can be drawn from the performed research:

- The addition of a 5% amount of zeolite with respect to asphalt mass does not significantly change the plastic deformation resistance and makes it possible to reduce the MA technological (laying) temperature by up to 30 degrees Celsius, which seems to be very important from ecological, economical, and pavement durability points of view.
- Based on the static indentation test, the best results in terms of resistance to permanent deformations are shown by the MA 8 PMB 25/55-60 mixture with natural zeolite and the MA 11 PMB 25/55-60 mixture with synthetic zeolite Na-P1.
- The dynamic indentation test revealed that both zeolites affect the deformation properties of the two MA 8 mixtures in a similar way; in the case of the MA 11 mixture with 35/50 bitumen, it is more beneficial to use the synthetic zeolite Na-P1, but for MA 11 with PMB 25/55-60 bitumen, both zeolites affect the behavior of the mixture in a similar way.
- According to Polish requirements based on the static indentation method, almost all tested MA mixtures with zeolite additives meet these requirements (one exception: MA 8 with PMB 25/55-60 bitumen); similarly, according to Swiss requirements based on the dynamic indentation method, almost all tested MA mixtures with zeolite additives meet these requirements (one exception: MA11 mixture with 35/50 bitumen).

## Figures and Tables

**Table 1:** The selected properties of both the tested bitumen.

Property	Test Method	Bitumen Type	
		35/50	PMB 25/55-60
Penetration at 25 °C [0.1 mm]	[31]	43	36
Softening point R and B [°C]	[32]	55.1	68.4
Fraass breaking point [°C]	[33]	−13	not tested
Elastic recovery at 25 °C [%]	[34]	not tested	79

**Table 2:** Chemical composition of clinoptilolite determined using XRF method.

Component	Content [%]
MgO	0.88
Al_2_O_3_	11.24
SiO_2_	76.93
K_2_O	3.94
CaO	3.77
TiO_2_	0.25
MnO	0.07
Fe_2_O_3_	2.71
Rb_2_O	0.02
SrO	0.04
ZrO_2_	0.02
Ag_2_O	0.08
BaO	0.05

**Table 3:** Static indentation test results after 30 min load.

MA Mixture	Zeolite Addition [%]	Results [mm]	
		35/50 Binder	PMB 25/55-60 Binder
MA 8	0Z	1.59 ± 0.22	0.94 ± 0.10
	5ZN	1.15 ± 0.12	1.04 ± 0.08
	5ZS	1.19 ± 0.14	1.09 ± 0.06
MA 11	0Z	1.62 ± 0.23	1.12 ± 0.17
	5ZN	1.82 ± 0.22	1.38 ± 0.04
	5ZS	1.87 ± 0.22	1.24 ± 0.15

**Table 4:** Increase in static indentation test results between 30 and 60 min loading.

MA Mixture	Zeolite Addition [%]	Results [mm]	
		35/50 Binder	PMB 25/55-60 Binder
MA 8	0Z	0.16 ± 0.03	0.11 ± 0.03
	5ZN	0.12 ± 0.03	0.10 ± 0.03
	5ZS	0.12 ± 0.01	0.12 ± 0.05
MA 11	0Z	0.17 ± 0.03	0.16 ± 0.04
	5ZN	0.20 ± 0.04	0.15 ± 0.04
	5ZS	0.18 ± 0.02	0.13 ± 0.02

**Table 5:** Dynamic indentation test results after 2500 cycles.

MA Mixture	Zeolite Addition [%]	Results [mm]	
		35/50 Binder	PMB 25/55-60 Binder
MA 8	0Z	0.94 ± 0.20	0.51 ± 0.11
	5ZN	1.45 ± 0.41	0.93 ± 0.20
	5ZS	1.54 ± 0.37	0.82 ± 0.11
MA 11	0Z	2.74 ± 0.46	0.84 ± 0.09
	5ZN	3.82 ± 0.49	1.26 ± 0.34
	5ZS	0.94 ± 0.20	0.51 ± 0.11

**Table 6:** Increase in dynamic indentation test results between cycles 2500 and 5000.

MA Mixture	Zeolite Addition [%]	Results [mm]	
		35/50 Binder	PMB 25/55-60 Binder
MA 8	0Z	0.28 ± 0.13	0.07 ± 0.05
	5ZN	0.41 ± 0.23	0.15 ± 0.10
	5ZS	0.48 ± 0.14	0.13 ± 0.03
MA 11	0Z	1.11 ± 0.19	0.17 ± 0.05
	5ZN	1.36 ±0.25	0.26 ± 0.17
	5ZS	1.13 ± 0.29	0.25 ± 0.08

## Data Availability

The raw data supporting the conclusions of this article will be made available by the authors on request.
